# Perinatal Outcomes and Associated Factors among women with hypertensive Disorders of Pregnancy Delivered in Jimma Zone Hospitals, Southwest Ethiopia

**DOI:** 10.4314/ejhs.v31i6.9

**Published:** 2021-11

**Authors:** Mesganew Amare, Adugna Olani, Habtamu Hassen, Bikila Jiregna, Nigusu Getachew, Sena Belina

**Affiliations:** 1 Department of Midwifery, College of Health Science, Mettu University, Ethiopia, misgeamare500@gmail.com; 2 School of Nursing and Midwifery, Institute of Health, Wollega University, Nekemte, Ethiopia; 3 Department of public health, Hossana Health Sciences College, Ethiopia; 4 Department of Midwifery, College of Health Science, Mettu University, Ethiopia; 5 Department of Health Policy and Management, Faculty of Public Health, Institute of Health, Jimma University, Ethiopia; 6 School of Nursing, Faculty of Health Science, Institute of Health, Jimma University, Ethiopia

**Keywords:** Perinatal outcomes, Hypertensive disorders of pregnancy, Jimma

## Abstract

**Background:**

Hypertensive disorders of pregnancy are multisystem diseases that increase the risk of adverse perinatal outcomes worldwide. It Led to early and late serious health consequence on the baby, with a significant proportion occurring in low-income countries. Hence the objective of this study was to determine perinatal outcomes and associated factors among women with hypertensive disorders of pregnancy delivered in Jimma zone hospitals.

**Method:**

A Facility based cross-sectional study design was employed from March to May 2020 on 211 hypertensive women delivered in the four randomly selected hospitals. The data were collected by reviewing medical record and face to face interview using consecutive sampling technique. Binary and multivariable logistic regression was performed to identify association.

**Result:**

Ninety-one (43.1%) of fetuses developed unfavorable perinatal outcome. Inability to read and write (AOR=2.5; 95% CI:1.03–6.17), being primipara (AOR=4.6; 95% CI:1.6–13.2) and multi-para (AOR=3.1; 95% CI:1.09–9.17), Lack of antenatal care visit (AOR=4.2; 95% CI:1.2–15.01), having preeclampsia (AOR=4.2; 95% CI:1.1–16.6) and eclampsia (AOR=5.8; 95% CI:1.2–26.2) and late provision of drug (AOR=3.9;95% CI:1.9–7.9) were independent factors.

**Conclusion:**

Pregnancy complicated with hypertensive disorders was associated with increased unfavorable perinatal outcomes. Preeclampsia and eclampsia, inability to read and write, primipara and multipara, lack of antenatal care and late provision of drug were factors associated with unfavorable perinatal outcomes.

## Introduction

Hypertension in pregnancy is the measurement of systolic blood pressure (BP) ≥ 140 mmHg or diastolic blood pressure ≥ 90 mmHg on two occasions at least six hours apart (1). According to International Society for the Study of Hypertension in Pregnancy (ISSHP) these disorders classified into five; gestational hypertension, chronic hypertension, preeclampsia, eclampsia and superimposed hypertension (2).

These disorders complicate 10% of all pregnancies and remain a leading cause of fetal morbidity and mortality worldwide (3). According to a study conducted on preeclampsia in low and middle-income countries, an estimated 72, 000 women and 500, 000 fetuses and newborns live endangered by preeclampsia and eclampsia each year. This result in almost 1600 life losses per day. Particularly sub-Saharan Africa and the Indian subcontinent account for 99% of these losses (4).

Research conducted on maternal and perinatal outcomes in nulliparous women complicated with pregnancy hypertension revealed as the majority of maternal and fetalneonatal complications occurred in induced hypertension particularly in severe preeclampsia (5). Among an estimated 2.6 million stillbirths that occur annually, 16% of them occurred in pregnancies complicated by pregnancy hypertension (6). Preeclampsia causes 1 in 10 late fetal and early neonatal deaths and 1 in 4 perinatal deaths (7).

Intrauterine growth restriction (IUGR), preterm birth, low birth weight, stillbirth, admission to neonatal intensive care unit due to these complications, and low APGAR score are common complications among babies born from the different spectrum of hypertensive disorder of pregnancy (HDP) (8–11). The burden is not limited to the pregnancy period, risks of mental health disorders and cognitive impairments increase in children and adults born from preeclamptic women (12). Even though there are variations in operationalizing, the prevalence of adverse perinatal outcomes of preeclampsia/eclampsia and pregnancy-induced hypertension in Ethiopia shows the variation in different parts of the country ranging from 46.5% to 66.4% (8,13,14).

The Ethiopian government and minister of health took different measures such as establishing maternal waiting homes, expansion of health facilities and provision of training for health professionals to reduce maternal and newborn mortality; considering the implementation of maternal, newborn and child health programs as a priority health agenda (15,16). Despite this, the proportion of the disease that causes severe risk consequence for life of the fetus and newborn than women increased by 154% from 2009 to 2013 and from 1.8% in 2011 to 5.7% in 2014 as revealed by research conducted in Addis Ababa (13) and North Shoa (17) respectively. Neonatal mortality also increased from 29 to 30 according to the 2019 mini Ethiopia Demographic and Health Survey (EDHS) (4). In Ethiopia, even if various studies were conducted on perinatal outcomes of HDP, little was done on factors associated with it which plays a vital role in the reduction of perinatal complications. The existing also showed controversies on some factors such as maternal age, antenatal care visit, types of HDP, maternal complication, and platelet count concerning their association with perinatal outcomes (9,18–20). Therefore, this study was aimed to determine perinatal outcomes and associated factors in women with HDP incorporating previously unstudied variables in our country such as HIV status and iron/folic acid supplementation.

## Methods and Participants

**Study area and period**: The study was conducted in Jimma Zone Hospitals which is located 356kms Southwest of Ethiopia from March 14 to May 16, 2020, among women with hypertensive disorders of pregnancy delivered in Jimma zone hospitals, Oromia regional state. The zone has eight governmental hospitals, two private hospitals, and 120 health centers. Out of the eight governmental hospitals, one is a referral hospital (JMC), three are general hospitals and four are primary hospitals. As the 2007 Ethiopia census reported it provides service for a total population of 2,486,155 out of which 1,235,628 are female.

**Study design and population**: A facility based cross-sectional study was performed. All women with hypertensive disorders of pregnancy delivered in the selected four Jimma zone hospitals who were diagnosed with any type of HDP and alive after delivery during the study period were included in the study.

**Sample Size Determination**: The sample size was determined using the Yamane formula (n= N/(1+N e^2^) by considering 420 population size, 95 confidence interval and 5% level of precision. This resulted to 205 sample size. Then adding 5% non-response rate, the final sample size was 215. Simple random sampling techniques were used to select the hospitals and the number of the average of the three consecutive months' cases of each hospital were taken from each hospital to estimate monthly available delivery cases. From the total of ten hospitals found in the Jimma zone, four (Jimma medical center, Agaro general hospital, Seka primary hospital, and Limu general hospital) were selected for the study. Finally, all eligible study participants were taken consecutively from each selected hospital.

**Data procedure and Instrument**: A structured questionnaire encompassing information related to socioeconomic and demographic, perinatal outcomes, and associated factors were retrieved first by record review in the maternity ward followed by a face to face interview with women who gave birth after they become stable. Cases of HDP were selected based on the diagnosis made by the health care providers. The data were collected by trained four BSc midwives and supervised by two BSc midwives who had previous data collection experience using pretested and structured “Afaan Oromo and Amharic” language version questionnaires that adapted from research conducted in the Amhara region and Wolaita Sodo teaching and referral hospital (9,13). The questionnaires were prepared in English then translated into the language of both Afan Oromo and Amharic versions then retranslated back to English by experts to ensure consistency of the instrument. Two days of training were given by the principal investigator which is focusing on the objective of the study to create a common understanding of the questionnaires. A pretest was conducted among 11 pregnant women in Shanan gibe hospital, after the pretest, the necessary correction was made and the reliability test was performed through Cronbach alpha (0.78).

**Study variables**: The dependent variable was perinatal outcomes and independent variables were socioeconomic and demographic factors, medical factors, service utilization factors, and Obstetrics factors.

**Data processing and analysis**: The collected data were checked for completeness and consistency manually before entry into the computer. Then, the questionnaires were coded and data were entered into Epi Info 7.2 version and exported to SPSS version 23 for analysis. Descriptive statistics like frequencies, cross tabulation, graphs, and percentages were employed. The goodness of fit data was checked with the Hosmer-Lemeshow test (p-value=0.35). Both binary and multivariable logistic regression analyses were employed to identify the candidate variables and contributing factors for perinatal outcomes among women with HDP respectively. Binary logistic regression analysis was used to identify the candidate variables for multivariable logistic regression at a p-value ≥ 0.25. Adjusted odds ratio (AOR) with 95%CI was used to determine the predictor of the outcome variable independently and to show the strength of an association p-value >0.5 was considered as statistically significant.

**Ethical consideration**: Ethical clearance was obtained from the Ethical Review Board of Jimma University, institute of health, College of Public Health, and Medical Science, and we also got a permission letter from the Jimma zone health office and each hospital's manager. The aim of the study was explained to the study participant. Each study participant was informed about the right to withdraw the consent and stop participation at any time without any form of prejudice. Privacy and confidentiality were maintained at each step of the study process. Written informed consent was obtained from the study participants to review their medical records and participate in the study.

## Results

**Socioeconomic and demographic characteristics**: Two -hundred- eleven women were interviewed in this study and yielded a 98.1% response rate. The mean ages of the respondents were 27.52 (± SD 5.657) years. Almost half (50.2%) of participants were referred from other health institutions. With regard to the participants' residence and average monthly family income, 116 (55%) were urban residents and 109 (51.7%) were with >1500–3000 ETB average monthly family income. Concerning the educational status, 76 (36%) of the participants attended elementary school and 50 (23.7%) were unable to read and write.

**Obstetrics characteristics and service utilization**: Ninety-seven (46%) of the participants were primipara. The current birth interval of ≥2 years was 73 (64%). Ninety-eight (46.4%) of participants gave birth by spontaneous vaginal delivery. Nearly two-thirds (64.7%) of the labor were spontaneous onset. The majority (89.1%) of participants had ANC visits and 117 (62.2%) started in the second trimester. Among those who had ANC visits, only two-fifths (39.9%) of them visited four times. Iron/folic acids were supplemented to the majority (83%) of participants.

**Type of hypertensive disorders of pregnancy and the time at which the drug is given**: Among 211 women with HDP, 117 (55.5%) of participants' encountered preeclampsia. 131 (62.1%) were treated early (before the severity sign and symptoms occurred).

**Maternal complications and perinatal outcomes**: Among 211 women with HDP, 80 (37.9%) encountered complications related to hypertension and HELLP syndrome (19.9%) accounted for the highest. Regarding perinatal outcomes, 91(43.1%) ended with an unfavorable outcome with 95% CI (36.3, 50.1). Among the unfavorable outcome's preterm birth, low birth weight, stillbirth, and IUFD encompass 64 (30.3%), 34 (16.1%), 13 (6.2%), and 4 (1.9%), respectively.

**Factors associated with perinatal outcomes**: Maternal age, educational status, parity, ANC visit, type of HDP, time of anti-hypertensive or/and anticonvulsant drug is given were significantly associated with the perinatal outcome by multivariable logistic regression with a p-value of <0.05.

The odds of having unfavorable perinatal outcomes were 2.5 times higher among hypertensive mothers who were unable to read/write compared to those mothers who were with the educational status of elementary school (AOR=2.5; 95% CI: 1.03–6.17). Similarly, the odds of having unfavorable perinatal outcomes were 4.6 AOR=4.6; (95% CI: 1.6–13.2) and 3.1 AOR=3.1; (95% CI: 1.09–9.17) times higher among mothers with HDP who were primipara and multipara respectively compared with grand multipara. Mothers who didn't attend ANC follow up were four times more likely AOR=4.2; (95% CI: 1.2–15.01) to have unfavorable perinatal outcomes than mothers who attend ANC follow up. Mothers diagnosed with preeclampsia and eclampsia were four times AOR=4.2; (95% CI: 1.1–16.6) and more than five times more likely AOR= 5.8; (95% CI: 1.2–26.2) to end up their pregnancy with unfavorable perinatal outcome compared to gestational hypertension respectively. Additionally, the odds of having Unfavorable perinatal outcomes were 3.9 times AOR=3.9; (95% CI: 1.9–7.9) higher among mothers with HDP who took drugs (anticonvulsant or/and anti-hypertensive) lately compared with mothers who took the drug early.

## Discussion

Hypertensive disorders of pregnancy disease complicate 10% of all pregnancies and remain a leading cause of both fetal and maternal morbidity and mortality worldwide. Fetal growth restriction, preterm birth, low birth weight, stillbirth, IFUD, and low APGAR score are common complications among babies born from a different spectrum of HDP (3,8–11). The study was attempted to assess the magnitude of perinatal outcomes and associated factors among women with hypertensive disorders of pregnancy delivered in Jimma zone hospitals. Our study pointed out that the overall prevalence of unfavorable perinatal outcomes was 43.1 % with 95% CI (36.3, 50.1) which is consistent with a cross-sectional study done in the Amhara region (46.5%) (13). However, it is lower than studies conducted in the Wolaita zone (57.8%) and the Tigray region (66.4%) (14,19). This might be due to variation in the operational definition of the dependent variable. On contrary, it is higher than study conducted in Jimma University Medical Center (23.13%) (20). This might be because of variation in service provided as our study encompasses primary and general hospitals in addition to this hospital.

Women who were unable to read/write were four times more likely to have an unfavorable outcome compared with those who were in elementary school. Consistently study done in the Amhara region suggests the more educated the educational status of mothers, the more favorable the perinatal outcome (13). This might be due to education promotes opportunities for better health-seeking behavior, lifestyle, and nutritional status.

Women who didn't attend ANC follow up were four times more likely to have unfavorable outcomes than those attending ANC. A similar result was noted by a study conducted in Wolaita Sodo teaching and referral hospital and other cohort studies (9,18). This might be due to ANC follow up promote an opportunity for early screening of HDP and timely initiation of treatment before further complications occurred. It also creates access to information related to nutrition and the severity sign of HDP.

Primipara and multipara women were more likely to have an unfavorable outcome. The unfavorable perinatal outcome was more than four times and three times more likely in primipara and multipara than grand multipara. This might be due to the repetitive experience of pregnancy in grand multipara makes them seek health service prior to the occurrence of complications. It might be also due to the severe consequence of HDP in primipara and multipara.

The unfavorable perinatal outcome was four times in preeclamptic and more than five times in eclamptic mothers than in gestational hypertension. Consistently, another study revealed that preeclampsia and eclampsia to increase the complication of fetal and neonatal outcomes than gestational hypertension (14). This could be due to differences in the severity of complications among each type of HDP.

Concerning the management of HDP, the time at which anti-hypertensive or/and the anticonvulsant drug is given found to have a significant association. Late provision of drugs was increased unfavorable outcomes by four times than early provision and consistent with research done in the Amhara region (13). This might be due to the fact that the early provision of drugs prevents the occurrence of advanced complications that could overwhelm maternal and fetal health.

In conclusion, pregnancy complicated with hypertensive disorders were ended up with high unfavorable perinatal outcome, predominantly preterm birth accounted for more than half of the total, while the remaining were low birth weight, stillbirth, and IUFD with the higher number respectively. Of the HDP preeclampsia and eclampsia, inability to read and write, being primipara and multipara, lack of ANC visit, and late provision of drugs were factors associated with unfavorable perinatal outcomes.

## Figures and Tables

**Figure 1:**
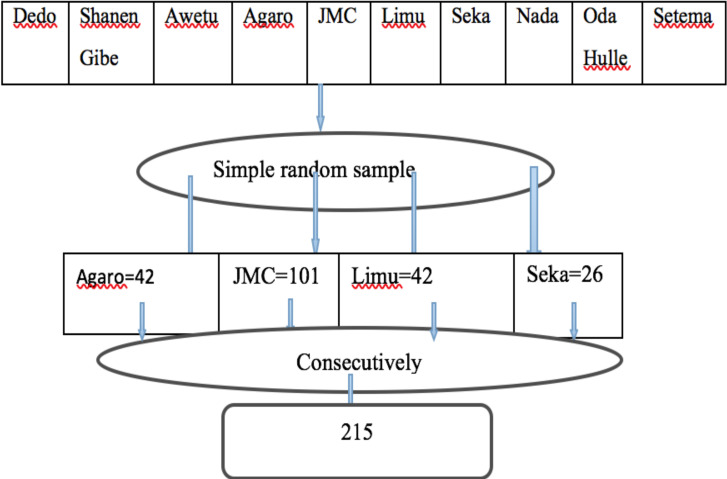
Schematic presentation of sampling technique to determine perinatal outcomes and associated factors in women with HDP delivered in Jimma zone hospitals, 2020

**Table 2:** Socioeconomic and demographic characteristics of study participants in Jimma zone hospitals, Southwest Ethiopia, 2020 (n=211)

Variable		Frequency	Percent
Maternal age	≤19	10	4.74
	20–34	179	84.83
	≥35	22	10.43
Marital status	Married	174	82.5
	Unmarried	15	7.1
	Divorced	12	5.7
	Widowed	10	4.7
Educational status	Unable to read and write	50	23.7
	Able to read and write	34	16.1
	Elementary school	76	36
	Secondary school	24	11.4
	College and above	27	12.8
Occupation	House wife	142	67.3
	Governmental	24	11.4
	Private	24	11.37
	Unemployed	11	5.2
	Others ^***^	10	4.7
Residence	Urban	116	55.0
	Rural	95	45.0
Referral status	Yes	106	50.2
	No	105	49.8
Average	≤1500	52	24.6
monthly income	>1500–3000	109	51.7
	>3000	50	23.7

Others *-Catholic, Adventist, Wakefeta; Others **-Tigre, Gurage, Yem, Silte Others ***-NGO, Student, Daily laborer

**Table 3:** Obstetrics characteristics and Service utilization of women with hypertensive disorders of pregnancy delivered in Jimma zone hospitals, Southwest Ethiopia, 2020

Variable		Frequency	Percent
Parity (n=211)	Primipara	97	46
	Multipara	77	36.5
	grand multipara	37	17.5
Current birth interval(n= 114)	≥2 years	73	64
	<2 year	41	36
	SVD	98	46.4
Mode of current delivery (n=211)	C/S before onset of labor	61	28.9
	Instrumental delivery	29	13.8
	C/S after onset of labor	23	10.9
Onset of labor(n=150)	Spontaneous	97	64.7
	Induced	53	35.3
ANC visit (n=211)	No	23	10.9
	Yes	188	89.1
Time of ANC started(n=188)	First trimester	58	30.9
	Second trimester	117	62.2
	Third trimester	13	6.9
Number of ANC visit (n=188)	One visit	12	6.4
	two visits	38	20.2
	three visits	63	33.5
	≥ four visits	75	39.9
Iron/folic acid supplemented (n=188)	No	32	17.0
	Yes	156	83.0

**Table 3:** Drugs given for treatment for women with hypertensive disorders of pregnancy delivered in Jimma zone hospitals, Southwest Ethiopia, 2020 (n=211)

Variable		Frequency	Percent
Type of HDP diagnosed	Preeclampsia	117	55.45
	Eclampsia	40	18.96
	Gestational	24	11.34
	Chronic	17	8.06
	Superimposed	13	6.16
Time of anti-hypertensive /anticonvulsant/ given	Early	131	62.1
	Late	80	37.9

**Table 4:** Maternal complications and perinatal outcomes among women with hypertensive disorders of pregnancy delivered in Jimma zone hospitals, Southwest Ethiopia, 2020 (n=211)

Variable		Frequency	Percent
Maternal complications	Uncomplicated	131	62.1
	Complicated	80	37.9
	HELLP syndrome	42	19.9
	Abruption placenta	19	9
	Pulmonary embolism	12	5.6
	DIC	6	2.8
	Acute renal failure	5	2.4
Perinatal outcomes	Favorable	120	56.9
	Unfavorable	91	43.1
Categories of unfavorable	Preterm birth	64	30.3
Outcome from the total	Low birth weight	34	16.1
	Still birth	13	6.2
	IUFD	4	1.9

**Table 5:** Results of multi-variable logistic regression analysis (n=211)

Variable		Perinatal outcomes		*AOR(95%C.I)*
			
		Favorable	Unfavorable	
Educational status	Unable to read and write	16	34	2.5(1.03–6.2)*
	Able to read and write	21	13	1(0.39–2.95)
	Elementary school	46	30	Ref
	Secondary school	17	7	0.36(0.11–1.2)
	College and above	20	7	0.6(0.21–1.96)
	Primipara	46	51	4.6(1.6–13.2)*
Parity	Multipara	47	30	3.1(1.09–9.2)*
	Grand multipara	27	10	Ref
ANC visit	No	4	19	4.2(1.2–15.0) *
	Yes	116	72	Ref
Types of HDP	Gestational	21	3	Ref
	Chronic	14	3	1.1(0.16–7.70)
	Preeclampsia	58	59	4.2(1.1–16.6) *
	Eclampsia	17	23	5.8(1.2–26.5)*
	Superimposed	10	3	1(0.14–8.49)
Time of drug given	Early	91	40	Ref
	Late	29	51	3.9(1.9–7.9) *

Ref= reference

* p-value <0.05

## References

[1] Dutta D (2013). DC Dutta's Textbook of obstetrics including perinatalogy and contraception.

[2] Brown MA, Magee LA, Kenny LC (2018). The hypertensive disorders of pregnancy: ISSHP classification, diagnosis & management recommendations for international practicetle. Pregnancy Hypertens.

[3] Wagner SJ, Barac S, Garovic VD (2007). Hypertensive Pregnancy Disorders : Current Concepts. J Clin Hypertens.

[4] Firoz T, Sanghvi H, Merialdi M von DP (2011). Pre-eclampsia in low and middle income countries. Best Pr Res Clin Obs Gynaecol.

[5] Fatemeh T, Marziyeh G, Nayereh G, Anahita G, Samira T (2010). Maternal and perinatal outcome in nulliparious women complicated with pregnancy hypertension. J Pak Med Assoc.

[6] Lawn JE, Blencowe H, Waiswa P, Amouzou A, Mathers C, Hogan D (2016). stillbirths: rates, risk factors, and acceleration towards 2030. Lancet.

[7] Hodgins S (2015). Pre-eclampsia as Underlying Cause for Perinatal Deaths : Time for Action.

[8] Asseffa NA DB (2019). Perinatal outcomes of hypertensive disorders in pregnancy at a referral hospital, Southern Ethiopia. PloS one.

[9] Wagnew M, Dessalegn M, Worku A, Nyagero J (2016). Trends of preeclampsia /eclampsia and maternal and neonatal outcomes among women delivering in addis ababa selected government hospitals, Ethiopia : a retrospective cross-sectional study. Pan Afr Med J.

[10] Mi A, Mm K, Fa S, Sa A, Ar M, Hossain A (2019). Evaluation of Maternal and Perinatal Outcome in Pregnancy Induced Hypertension. Med Col J.

[11] Mersha AG, Abegaz TM SM (2019). Maternal and perinatal outcomes of hypertensive disorders of pregnancy in Ethiopia: systematic review and meta-analysi. BMC Pregnancy Childbirth.

[12] Tuovinen S, Eriksson JG, Kajantie E, Lahti J, Pesonen A, Heinonen K (2013). Maternal hypertensive disorders in pregnancy and self-reported cognitive impairment of the offspring 70 years later: the Helsinki Birth Cohort Study. Am J Obs Gynecol.

[13] Melese MF, Badi MB, Aynalem GL (2019). Perinatal outcomes of severe preeclampsia / eclampsia and associated factors among mothers admitted in Amhara Region referral hospitals, North West Ethiopia, 2018. BMC Res Notes.

[14] Aimakhu O MA (2017). Effect of pregnancy induced hypertension on adverse perinatal outcomes in Tigray regional state, Ethiopia: a 3 prospective cohort study. BMC Pregnancy Childbirth.

[15] Federal Ministry of Health (2016). National strategy for newborn and child survival in Ethiopia.

[16] United States Agency for international development (2013). Promising Practices in Maternal and Newborn Health and Family Planning and Reproductive Health.

[17] Getachew Y, Derbew M, Mariam DH (2015). patterns of hypertensive disorders of pregnancy and associated factors at debre berhan referral hospital, north shoa. Ethiop J Reprod Heal.

[18] Subki AH, Algethami MR (2018). Prevalence, Risk Factors, and Fetal and Maternal Outcomes of Hypertensive Disorders of Pregnancy: A Retrospective Study in Western Saudi Arabia. Oman Med J.

[19] Obsa M, Wolka Woticha E, Girma Weji B, Kassahun Dessu B, Gebremskel Girmay B, Dendir Wolde (2019). Neonatal and Fetal Outcomes of Pregnant Mothers with Hypertensive Disorder of Pregnancy at Hospitals in Wolaita Zone, Southern Ethiopia. J Midwifery Reprod Heal.

[20] Assefa F, Siraneh Y, Taye A (2020). Outcome of hypertensive disorders of pregnancy and associated outcome of hypertensive disorders of pregnancy and associated factors among pregnant women admitted to abstract method. Ethiop J Reprod Heal.

